# Dr. Adams *in Answer to Dr. Curry*

**Published:** 1818-06

**Authors:** 


### *For the London Medical and Physical Journal.*

### Dr. ADAMS *in Answer to Dr. Curry.*

AS you have done me the honour to send me a letter, with your own signature, before it was given to this Journal, accompanied with Dr. Morgan's Treatise, not to answer you with my signature might appear disrespectful.

You first apologise for not complying with a reasonable request, in producing Dr. Morgan's Dissertation. This apology was unnecessary, or came too late; when, in consequence of your former delay, so many passages had been quoted from the Dissertation, as to show that the writer had already met with the work. You next "rely on my candour for a faithful statement of its contents." I should rather rely on your candour and better judgment, to inform me, whether I have omitted to notice any passage in which Dr. Morgan more pointedly states his doctrine than in those I

have transcribed; and, whether those passages can be said to contain Mr. Hunter's theory of pus. In the transcript already made, I seem to have done more than fair controversy demands, because no one is expected to prove a negative, but only to answer the arguments of the affirmer.

If you mean to suggest, that I "consider Dr. Hunter's denial of the Boerhaavian theory a proof that he taught the formation of pus by a secretory process," or that "the mere circumstance of John Hunter being an assistant to his brother is to deprive the latter of every claim to discovery,"—I must have expressed myself more obscurely than I am generally accused of doing.

Having waited more than a month to see if any one would point out the passages in Dr. Morgan's Dissertation, in which he gives any intelligible doctrine of the process of suppuration and ulceration, I procured the work from Bolt-court, and took much pains to select those passages which, in my reading, seemed to bear the nearest to the question; and, after a fair consideration of all I could collect on the subject, Dr. Morgan appeared to me merely to have hazarded a loose conjecture, or to have entertained some confused notion of the Hunterian doctrine, which he was unable to explain, or, of course, to render intelligible.

Till, therefore, I can understand what Dr. Morgan's opinions are, (beyond a mere conjecture concerning "*puris confectionem œconomiæ animalis opus esse secretioni maxime analogum aut revera fluidi peculiaris secretionem ex sanguine vivi, jactam, virtute vasorum ex debito gradu inflammationis præcuntis huic accommodatorum,*) I do not think myself called upon to prove, that he did NOT precede Mr. Hunter in the doctrine of what he calls *pyopoiesis.*

If Dr. Morgan had any distinct notions of the only doctrine of suppuration and ulceration that has been of late years attended to, I will thank you to point them out, and still more to reconcile them with his adherence to those doctrines of Boerhaave, Galen, and Celsus, by which he endeavours to illustrate, if not support, his own. Until this be done, I must consider Dr. Morgan's as a happy, though very crude, conjecture, much behind those discoveries which were made sixteen years earlier than the date of his Thesis, and which, from that time, appear to have excited discussion in all the London schools of medicine.

*Hatton-garden; May* 7, 1818.

*** The papers referred to being contained in the present volume, (pages 89 for Feb., 285 for April, and 353 for May,) it is presumed that the reader, who interests himself in the question, will re-peruse each; on which account, a transcript of the parts alluded to has been deemed unnecessary.

